# Caloric Intake in Renal Patients: Repercussions on Mineral Metabolism

**DOI:** 10.3390/nu13010018

**Published:** 2020-12-23

**Authors:** Angela Vidal, Rafael Ríos, Carmen Pineda, Ignacio López, Ana I. Raya, Escolástico Aguilera-Tejero, Mariano Rodríguez

**Affiliations:** 1 Department Medicina y Cirugia Animal, University of Cordoba, 14071 Cordoba, Spain; v92vicaa@uco.es (A.V.); rafariosvaro@me.com (R.R.); v32pimac@uco.es (C.P.); l02lovii@uco.es (I.L.); v82rabea@uco.es (A.I.R.); 2 Maimonides Biomedical Research Institute of Cordoba (IMIBIC), University of Cordoba, 14004 Cordoba, Spain; marianorodriguezportillo@gmail.com

**Keywords:** diet, calories, mineral metabolism, kidney disease

## Abstract

The aim of this paper is to review current knowledge about how calorie intake influences mineral metabolism focussing on four aspects of major interest for the renal patient: (a) phosphate (P) handling, (b) fibroblast growth factor 23 (FGF23) and calcitriol synthesis and secretion, (c) metabolic bone disease, and (d) vascular calcification (VC). Caloric intake has been shown to modulate P balance in experimental models: high caloric intake promotes P retention, while caloric restriction decreases plasma P concentrations. Synthesis and secretion of the phosphaturic hormone FGF23 is directly influenced by energy intake; a direct correlation between caloric intake and FGF23 plasma concentrations has been shown in animals and humans. Moreover, in vitro, energy availability has been demonstrated to regulate FGF23 synthesis through mechanisms in which the molecular target of rapamycin (mTOR) signalling pathway is involved. Plasma calcitriol concentrations are inversely proportional to caloric intake due to modulation by FGF23 of the enzymes implicated in vitamin D metabolism. The effect of caloric intake on bone is controversial. High caloric intake has been reported to increase bone mass, but the associated changes in adipokines and cytokines may as well be deleterious for bone. Low caloric intake tends to reduce bone mass but also may provide indirect (through modulation of inflammation and insulin regulation) beneficial effects on bone. Finally, while VC has been shown to be exacerbated by diets with high caloric content, the opposite has not been demonstrated with low calorie intake. In conclusion, although prospective studies in humans are needed, when planning caloric intake for a renal patient, it is important to take into consideration the associated changes in mineral metabolism.

## 1. Introduction

Caloric intake is known to influence the development and progression of renal disease. In general, diets rich in calories (due to increased fat or to increased carbohydrates) are deleterious for the kidneys. Diets with high fat content have been reported to cause renal damage in non-obese animals that did not develop type 2 diabetes, suggesting a direct effect of high fat/high calorie intake on the kidney [1]. Podocyte injury secondary to inflammasome activation [2] and down-regulation of the sirtuin type 1 (Sirt 1)–adiponectin axis [3] seem to be involved in nephrotoxicity associated with elevated caloric intake. High caloric intake is also known to alter the expression of genes related to cytoskeleton remodelling and to induce renal fibrosis [4]. In addition to promoting direct kidney damage, high caloric intake also affects renal disease indirectly (through the development of obesity and type 2 diabetes). Obesity may influence the progression of chronic kidney disease (CKD) due to its effects on renal hemodynamics that result in hyperfiltration, increased glomerular pressure, and podocyte damage [5,6]. Type 2 diabetes causes renal disease secondary to microvascular changes within the kidney and metabolic abnormalities leading to thickening of the glomerular basement membrane, expansion of the mesangial matrix, nodular glomerulosclerosis, arteriolar hyalinosis, endothelial dysfunction, and podocyte injury [7]. 

Conversely, caloric restriction (CR) has been shown to protect the kidneys and preserve renal function [8]. CR, defined as a reduction in daily caloric intake below the amount that would be consumed ad libitum without incurring in malnutrition, can be achieved by a variety of means including (a) reducing the total amount of food that is eaten daily, (b) changing the diet composition by decreasing energy-dense nutrients, (c) modifying eating habits with intermittent periods of fasting, or (d) by medical interventions, such as bariatric surgery [9,10]. The variety of protocols that may lead to CR makes it difficult to compare the outcomes. However, most experimental studies with animals and the few studies that have investigated the relationship between CR and mineral metabolism tend to use a reduction in the total amount of food that averages around 30% of daily intake [11]. Short-term CR decelerates renal aging by preventing increases in glomerular volume, decreasing fibrosis, and reducing senescence-associated β-galactosidase staining in aged rats [12]. Long-term CR was found to exert beneficial effects in rodents at different stages (from early onset to advanced kidney failure) of renal disease [13]. Although the exact mechanisms for the renoprotective effect of CR are not known some of the reported changes observed after CR that may be beneficial for the kidney include improving mitochondrial biogenesis and DNA repair, reducing inflammation and oxidative stress [13], increasing AMP-activated protein kinase and Sirt1, increasing autophagy, reducing molecular target of rapamycin (mTOR) [13,14,15], and lowering the soluble form of the receptor for advanced glycation end products (RAGE) [16]. CR has also been reported to ameliorate renal function resulting in decreased concentrations of serum urea nitrogen, serum creatinine, and urine protein and to increase survival rate in rodents with CKD [17]. 

Adjusting caloric intake in the renal patient is not straightforward, and the recommendations are related to the stage of kidney disease. CKD is commonly classified in 5 stages, according to the reduction in glomerular filtration rate (GFR): stage 1 (GFR > 90 mL/min), stage 2 (GFR = 60–89 mL/min), stage 3 (GFR = 30–59 mL/min), stage 4 (GFR = 15–29 mL/min), and stage 5 (GFR < 15 mL/min) [18]. In earlier stages (CKD stages 1 and 2), most patients (especially the obese population with type 2 diabetes) would benefit from CR. As renal deterioration progresses, patients commence losing appetite and weight. Weight loss may start early in the course of renal disease and is associated with progression of CKD and mortality [19]. Defective hypothalamic appetite regulation promotes anorexia in uremic patients [20]. Moreover, poor appetite is often aggravated by dietary restrictions that are primarily aimed at reducing phosphorus (P) and potassium intake but that negatively affect food palatability. Thus, when patients are losing weight (CKD stages 3 to 5) dietary strategies are usually shifted towards preservation of caloric intake. Nonetheless, measures to prevent a deleterious effect of high caloric intake on CKD should be installed early in the progression of the disease.

Renal disease is associated with changes in mineral metabolism, that are known collectively as chronic kidney disease/metabolic bone disease (CKD-MBD), and that include secondary hyperparathyroidism, changes in parameters of mineral metabolism, bone disorders, and extraskeletal calcification. Changes in calciotropic hormones occur early in the course of CKD. Secretion of fibroblast growth factor 23 (FGF23) and parathyroid hormone (PTH) are increased to prevent accumulation of P and hypocalcaemia. The decline in effective renal mass results in reduction in calcitriol production and therefore circulating calcitriol concentrations are low in CKD [21]. When deterioration of renal function progresses, the kidneys are not able to handle a normal P load, even with elevated FGF23 and PTH, and hyperphosphatemia ensues. Elevated concentration of extracellular P is a major factor in the development of VC [22]. In addition, changes in mineral metabolism are often accompanied by bone disease [23]. Emerging data indicate that both excessive caloric intake and reduced caloric intake may promote bone disorders [24,25,26,27], although their influence on CKD-MBD has not been sufficiently explored [28].

Accumulating evidence demonstrates an inter-relationship between energy metabolism and mineral metabolism [29,30,31]. Thus, in addition to the factors that have been classically considered as target for the control of CKD-MBD (such as calcium (Ca), P, and PTH), it may also be necessary to consider integrating adjustment of caloric intake for a more comprehensive control of mineral disorders in the CKD-MBD population.

The aim of this paper is to review current knowledge about how calorie intake influences mineral metabolism focussing on four aspects of major interest for the renal patient: (a) P handling, (b) FGF23 and calcitriol synthesis and secretion, (c) metabolic bone disease, and (d) VC.

## 2. Caloric Intake and P Handling 

Decreased renal function results in impaired P handling leading to P retention and eventually to increased plasma P concentrations with secondary hyperparathyroidism [32]. Elevated P load, as reflected by high urinary P, is not only an outcome of poor renal function but also promotes the progression of kidney disease [33]. Thus, control of P intake is paramount in the renal patient, especially in the more advanced stages (CKD stages 4 and 5) of the disease.

**Figure 1:**
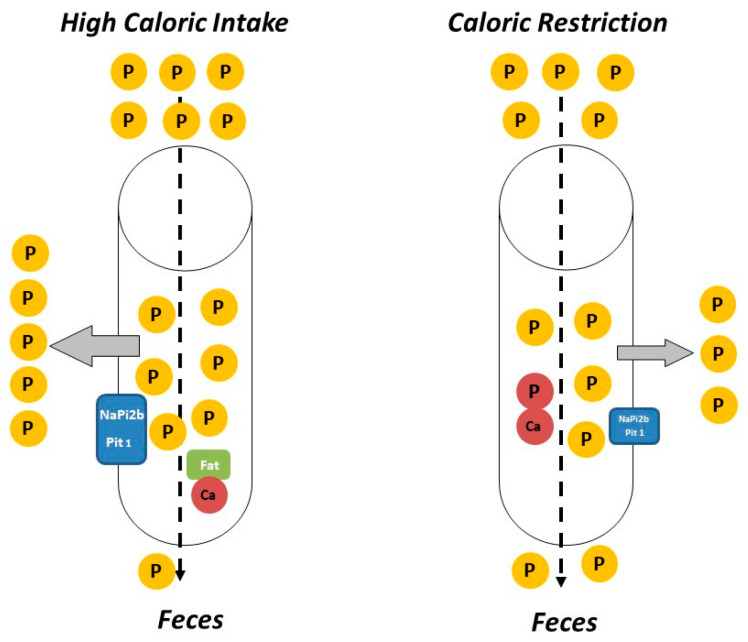
Caloric intake (especially fat intake) influences phosphorus (P) balance. High caloric intake (**left**) usually is associated with consumption of foods with higher P content than calorie-restricted foods (**right**). In addition, intestinal absorption of P is generally more efficient in calorie-rich/fat-rich food due to the formation of fat–calcium (Ca) soaps and to upregulation of P transporters (NaPi2b and Pit1). Thus, calorie/fat restriction tends to decrease P balance and plasma P concentrations.

P intake is related to the caloric content of food in several ways. On the one hand, high caloric intake is usually related to frequent consumption of energy-dense foods (e.g., fast food) that are typically rich in P, not only because of their intrinsic P content but also because P is widely used as a food additive. It is important to note that the inorganic P added to processed foods is more readily absorbed than the organic P naturally contained in foodstuffs [34]. Information about the actual P content of food is scant and fast food is likely to represent a hidden source of dietary P intake [35]. Additionally, a high fat content in the diet has been reported to increase P digestibility [36,37], further aggravating P load. The influence of dietary fat on intestinal P absorption seems to be secondary to the formation of Ca soaps in the intestinal lumen. Trapping of Ca in Ca soaps decreases the generation of insoluble Ca-P complexes, thus allowing more P ions to be free and able to be absorbed [36]. Diets rich in fat may also enhance paracellular transport of P by increasing intestinal permeability and they have been reported to upregulate P transporters NaPi2b and Pit1 in the small intestine [37] (Figure 1). The impact of intestinal absorption of P on serum P levels is critical at the time CKD has progressed to stages 4–5 when the kidney filtration of P is very limited and the ability to excrete P in the urine is markedly impaired despite the phosphaturic stimulus of FGF23 and PTH. Data obtained in experimental animals indicate that high caloric intake due to increased fat in the diet would contribute to P retention in individuals with reduced renal function. It has been shown that feeding high-fat diet leads to P retention when either renal function is decreased or when the P content of the diet is increased. Moreover, a tendency to increased plasma P concentration was identified both in rats with normal and decreased renal function after high-fat feeding [31].

The impact of CR on P homeostasis is largely unknown, although the scant available data indicate that decreasing caloric intake would help to reduce P load. Low calorie foods (e.g., vegetables) tend to have lower P content than energy-dense foods and P-containing additives are less commonly used in these types of food. Moreover, the mere fact of reducing food intake would also result in a decrease in total P intake independent of the P content of the foodstuffs. The reduction in caloric intake is also likely to further decrease intestinal P absorption (Figure 1). A recent study in non-obese rodents has shown that animals subjected to CR had lower plasma P concentrations than their non-calorie restricted counterparts even though the daily P intake was identical in both groups. Moreover, the calorie restricted animals showed a tendency to decreased P balance [11]. 

Besides P, other minerals that are relevant in the CKD patient are Ca and magnesium (Mg). However, information about the influence of caloric intake on these minerals is sparse. CKD patients tend to have low Ca and Mg levels. High-fat diets might contribute to aggravate hypocalcaemia, because fat has been shown to reduce intestinal absorption of calcium [38]. Reduction in food intake may lead to decreased ingestion of Ca and Mg; thus, when implementing CR, care should be taken to ensure appropriate Ca and Mg intake. Increasing Mg intake can be beneficial in CKD, because Mg improves glycaemic control [39] and protects against VC [40,41]; however, Mg may also contribute to increase body weight and adiposity [42].

In conclusion, in addition to the benefits gained by their intrinsic lower P content and the less frequent presence of P containing additives in low-calorie foods, current evidence indicates that even for a fixed P intake CR would help to decrease P load. Thus, reducing calorie intake could be an additional strategy for controlling plasma P in CKD patients that are in earlier stages of the disease (stages 1 to 3). This nutritional strategy may help to obviate the need of implementing pharmacological therapies (like P binders) or to retard their prescription. In patients with more advanced CKD, in which P control is still more important, the use of CR would be limited by the development of weight loss/malnutrition.

## 3. Caloric Intake, FGF23, and Calcitriol

### 3.1. FGF23

FGF23 is a phosphaturic hormone secreted by osteocytes/osteoblasts that plays a major role in the regulation of mineral metabolism [43]. Interaction of FGF23 with its receptor, FGFR1c, and its co-receptor, α-klotho, increases phosphaturia by inducing internalization of the sodium-dependent P transporters (NaPi2a and NaPi2c) [44]. Synthesis and secretion of FGF23 is regulated mainly by dietary P and increased P balance is thought to be the main stimulus for FGF23 secretion [45]. In addition, FGF23 production is under hormonal control by calcitriol, which increases FGF23 synthesis [46]. Moreover, PTH [47], inflammation [48], and iron deficiency [49] have also been reported to influence FGF23. 

Epidemiological studies have suggested that FGF23 might be regulated by calorie intake. FGF23 has been shown to be elevated in obese people [50] and increased caloric intake has been identified as a potential predictor of plasma FGF23 concentrations [51]. Experimental work has shown that feeding hypercaloric diets to rodents results in a consistent increase in plasma and bone FGF23 [1,31]. In these animals, a decrease in renal α-klotho expression was also observed, and thus, it was speculated that the increase in FGF23 might be secondary to the reduction in renal α-klotho. In calorie-replete animals the decrease in α-klotho would create a state of FGF23 resistance in which more FGF23 is needed to preserve phosphaturia. Further studies in non-obese rats subjected to CR have shown a reduction in circulating concentrations of FGF23, which is accompanied by an increase in renal α-klotho [11], this α-klotho abundance would require less FGF23 to maintain P homeostasis in calorie-restricted animals. Thus, regulation of renal α-klotho by caloric intake could influence FGF23 synthesis and secretion. Studies in humans also support the role of reducing caloric intake on FGF23, e.g., a decrease in circulating FGF23 concentrations has been reported in patients subjected to bariatric surgery [52].

In addition to these in vivo studies, factors involved in energy metabolism have been reported to directly regulate FGF23 production by bone cells in vitro. Increases in both insulin and AMP-activated kinase (AMPK) have been shown to decrease FGF23 synthesis [53,54]. A recent study has demonstrated that FGF23 secretion by bone cells cultured in vitro is directly regulated by nutrient availability in the medium. Cells cultured in medium with high glucose consistently synthesized more FGF23 than cells cultured in medium with low glucose. Moreover, energy availability seemed to override the regulatory effect of P on FGF23, because cells incubated with high P concentrations only increased FGF23 expression when cultured in medium with high glucose. It is interesting to note that treatment with drugs that mimic CR (the mTOR inhibitors rapamycin and everolimus) was also able to down-regulate FGF23 expression even in the presence of high glucose in the medium [55].

The mechanisms involved in the regulation of FGF23 by energy intake have been studied, but they are not completely understood. A study demonstrated that AMPK activation decreases FGF23 and that the effect of AMPK on FGF23 was mediated by down-regulation of the Ca channel Orai1 involving store-operated Ca entry (SOCE) [54]. Another study reported that insulin suppresses the production of FGF23 by inhibition of FOXO1 transcription factor [53]. The influence of the mTOR signalling pathway on the regulation of FGF23 by energy availability was recently explored. Two mTOR complexes, mTORC1 and mTORC2, are known, but only mTORC1 is rapamycin sensitive. mTORC1, which is suppressed by energy deprivation (low ATP-to-AMP ratio), is a downstream target of AMPK [56]. Both AMPK and rapamycin have an inhibitory effect on mTOR, but the fact that rapamycin by itself is able to decrease FGF23 production focus the signalling of energy-sensing that regulates FGF23 on mTOR. It is interesting to note that a tendency to decreased FGF23 has also been reported in mice treated with rapamycin [57] lending further support to the involvement of the mTOR pathway in FGF23 production. mTORC1 signalling can also modulate SOCE and mTORC1 inhibition by rapamycin has been shown to suppress STIM1, a protein necessary for SOCE activation [58]. A decrease in STIM1 expression in osteogenic cells incubated with rapamycin and everolimus has also been reported [55]. Thus, both AMPK activation, through orai1 inhibition, and mTORC1 inhibition, through STIM1 down-regulation, can modulate SOCE activation resulting in a decrease in FGF23 production. The involvement of the mTOR pathway in energy-regulation of FGF23 production by bone cells may help to harmonize the apparent contradiction of decreased FGF23 in response to both insulin (that is associated with energy repletion) [53] and low glucose with subsequent activation of AMPK (which is activated by energy depletion) [54]. Moreover, it may provide explanation to the paradox of insulin, which inhibits AMPK [59], having the same effect on FGF23 that AMPK. Both, insulin signalling, through the phosphatidylinositol 3-kinase (PI3K/Akt) pathway, and mTOR inhibition by rapamycin (or by energy restriction) are able to inactivate FOXO1 [53,60]. Thus, it has been proposed that mTOR may be a central molecule in the regulation of FGF23 by energy availability, since it integrates two signalling pathways that are dependent on insulin and energy availability, respectively [55]. In the context or renal patients subjected to renal transplantation that are treated with mTOR inhibitors (e.g., everolimus), it would be important to study how these drugs influence FGF23.

**Figure 2:**
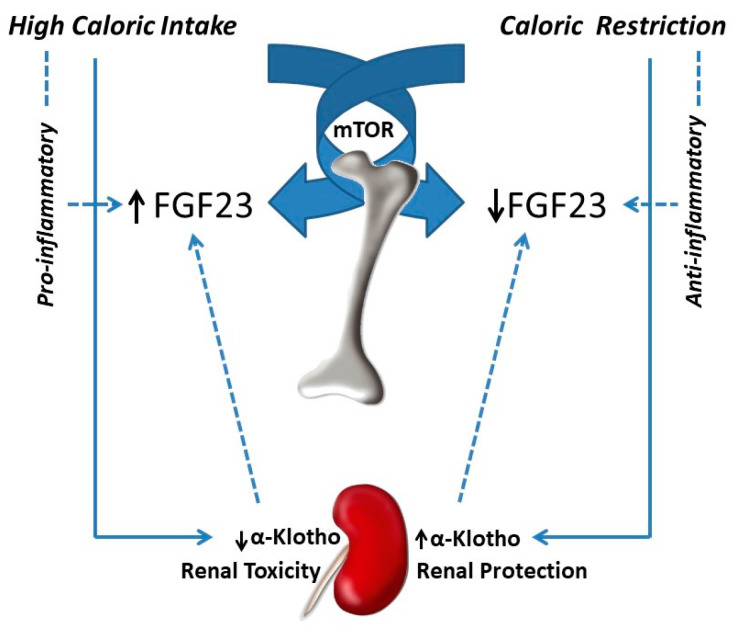
Fibroblast growth factor 23 (FGF23) regulation by caloric intake. High caloric intake increases FGF23, while caloric restriction decreases FGF23. The mechanisms of FGF23 regulation by caloric intake include a direct effect on bone, mediated through the mTOR signalling pathway, and indirect mechanisms, mainly through renal α-klotho regulation. Other indirect mechanisms that may influence FGF23 comprise the inflammatory/anti-inflammatory action of high vs. low caloric intake.

In conclusion, two main mechanisms seem to be implicated in the regulation of FGF23 by caloric intake: indirect mechanisms, involving regulation of renal α-klotho expression, and direct mechanisms, which affect bone cell metabolism through the mTOR pathway. In addition, other factors associated with differential energy intake, such as inflammation, may also play a role in the regulation of FGF23 by the caloric content of the diet (Figure 2).

FGF23 is an important metabolite in kidney patients not only for its ability to promote P excretion but also because increased levels of FGF23 have been reported as a risk factor of cardiovascular mortality [61]. Actually, the relationship between high FGF23 and mortality is not restricted to patients with kidney disease but has also been identified in the general population [62,63]. Thus, it is important to maintain FGF23 concentration within a physiologic range. On the other hand, some studies have demonstrated that, in the presence of significant renal disease, high levels of FGF23 may be necessary to control mineral metabolism and that an excessive reduction in FGF23 could be detrimental [64,65]. From a clinical perspective, given the fact that the increase in FGF23 concentrations is one of the earliest biochemical changes that can be identified in renal patients and that it precedes the increase in plasma P, the modulatory effect of the caloric content of the diet on FGF23 may be very relevant. In this context, the downregulation of FGF23 by reduced caloric intake opens a new window to control FGF23 without interfering with P balance (in fact, helping to maintain a favourable P balance, which would prevent further renal deterioration by decreasing the exposure of distal tubular cells to P). 

### 3.2. Calcitriol

Vitamin D is an essential micronutrient mainly involved in bone and mineral metabolism. Vitamin D is synthesized in the skin or ingested with the diet as cholecalciferol/ergocalciferol, which subsequently is metabolized in the liver to 25(OH)-cholecalciferol/ergocalciferol (calcidiol). Calcidiol is further hydroxilized in the kidney to produce 1,25(OH)_2_-cholecalciferol/ergocalciferol (calcitriol), which is the major active metabolite of vitamin D [66].

Calcitriol production by the kidneys decreases as a consequence of the progression of renal disease and patients with CKD typically have decreased plasma calcitriol concentrations [67,68]. Low vitamin D concentrations have been associated with cardiovascular disease [68], inflammation [69], endothelial dysfunction [70], and increased risk of bone fractures [71]. In the CKD-MBD patient, decreased vitamin D levels may have a major influence in the development of skeletal disorders (including fractures) and in long-term survival [72,73]. Thus, vitamin D replacement therapy (using calcitriol or calcitriol-analogues) is often necessary in patients with CKD-MBD, although this treatment may have significant side effects (extraskeletal calcifications) [67].

The relationship between caloric intake and vitamin D levels is not well documented and most available data relate vitamin D (usually calcidiol) with body weight. Obesity, insulin resistance, and type 2 diabetes are associated with low vitamin D levels in both humans and rodents [74,75,76,77,78]. Moreover, the bioavailability of vitamin D is decreased in obese individuals [79]. 

Experimental studies in rats have shown that feeding diets with high caloric concentration results in decreased plasma calcitriol even in animals that do not experience an increase in body weight [31]. When comparing rats fed high and normal fat diets with identical vitamin D content, plasma calcitriol was significantly lower in rats fed high fat [1,31]. The influence of dietary fat on intestinal absorption of vitamin D is somewhat controversial but diets with high fat content have been reported to increase vitamin D absorption by the intestine [80]; thus, decreased absorption of vitamin D is unlikely in rats fed high fat. Moreover, plasma calcidiol concentrations were not decreased in rats fed high fat diets [31]. Since calcidiol is the metabolite that best reflects nutritional status for vitamin D [81], the origin of the decreased plasma calcitriol cannot be attributed to decreased vitamin D intake. 

The information relating vitamin D and CR is sparser. A recent study has identified an increase in calcidiol in obese adults subjected to ketogenic diet-induced weight loss [82]. In animal studies, plasma calcitriol concentrations have also been reported to increase in non-obese rats subjected to CR [55].

In summary, epidemiological data demonstrate an inverse relationship between vitamin D status (as assessed by circulating calcidiol concentrations) and body weight/glycaemic control. Although plasma calcidiol has been shown to accurately reflect vitamin D intake/skin synthesis, in the renal patient what is critical is the decrease in calcitriol production by the kidney. As discussed above, experimental studies in rats have shown that high-fat diets decrease calcitriol concentrations while CR increases calcitriol concentrations [31,55]. 

**Figure 3:**
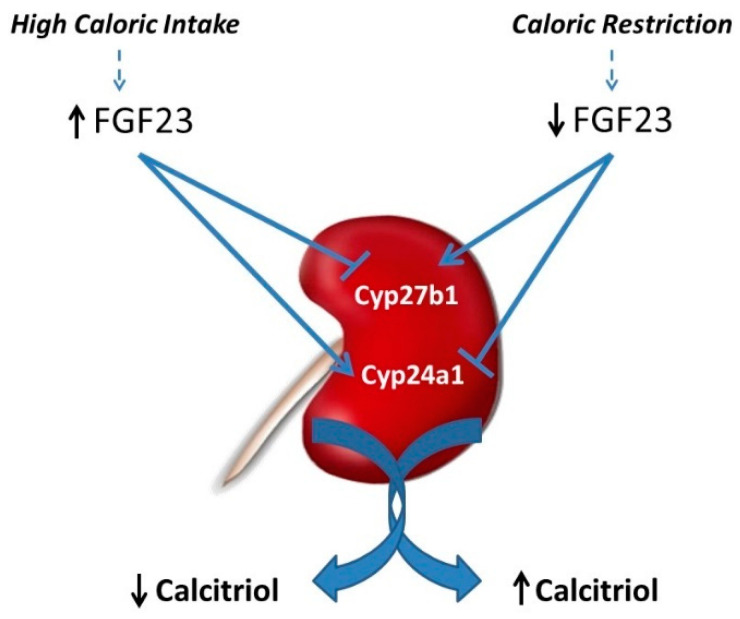
Calcitriol regulation by caloric intake is secondary to changes in fibroblast growth factor 23 (FGF23). The increase in FGF23 after high caloric intake down-regulates Cyp27b1 (involved in calcitriol synthesis) and upregulates Cyp24a1 (that metabolizes calcitriol) leading to decreased calcitriol concentrations. Conversely, the decrease in FGF23 after calorie restriction upregulates Cyp27b1 and down-regulates Cyp24a1 resulting in increased calcitriol concentrations.

The most likely explanation for the regulation of calcitriol after modifying caloric intake is related to changes in FGF23. FGF23 down-regulates calcitriol production by the kidney through the inhibition of the enzyme that converts calcidiol to calcitriol: Cytochrome P450 Family 27 Subfamily B Member 1 (Cyp27b1), and by stimulation of the enzyme 24-hydroxylase: Cytochrome P450 Family 24 Subfamily a Member 1 (Cyp24a1) that catabolizes calcitriol [83]. Thus, the elevated FGF23 concentrations found after feeding high calories would down-regulate Cyp27b1 and upregulate Cyp24a1 to decrease calcitriol, while the reduced FGF23 observed after caloric restriction would upregulate Cyp27b1 and down-regulate Cyp24a1 to increase calcitriol (Figure 3). 

In conclusion, based on the results of animal studies, reduction in caloric intake, in addition to aid in the control of FGF23, may help to prevent the decrease in calcitriol concentrations in CKD-MBD patients. As it has been already discussed for P and FGF23, the effect of CR on calcitriol would be beneficial along the whole range of CKD stages but, for practical reasons, would be more likely applicable to patients in early stages in which decreased appetite and weight loss are not yet a concern.

## 4. Caloric Intake and Metabolic Bone Disease (MBD)

Bone abnormalities are frequent in patients with CKD and represent an important contributor to decreased quality of life because of the risk of fractures and reduced mobility [84]. Renal patients have fragile bones as a consequence of decreased bone mineral content and abnormal bone architecture. MBD encompasses alterations in bone structure characterized by abnormalities in the rate of turnover, mineralization, and bone volume [85]. Uremic toxins influence bone quality in CKD patients [86], and MBD is a more complex entity than classic osteoporosis [87]. Evidence suggests that patients with CKD have an elevated risk of bone fractures, especially in end-stage renal disease because of the high incidence of MBD [88]. The altered mechanical properties of uremic bone have been demonstrated in animal models with experimental CKD, in which elasticity of femoral bones evidenced an inverse correlation with kidney function [89]. In humans, the Kidney Disease: Improving Global Outcomes (KDIGO) guidelines encourage monitoring bone mass in the CKD patient [84], and adjusting caloric intake may help to prevent bone loss.

Caloric intake has a profound influence on bone parameters. In general, high caloric intake results in increased bone mass, while low caloric intake decreases bone mass. The effect of caloric intake on bone is in part indirect and related to changes in body weight. The relationship between obesity and bone metabolism is complex and has not yet been fully elucidated. Obesity was traditionally thought to have protective effects on the skeleton. However, this assertion has recently been challenged with studies demonstrating that obesity is associated with a lower risk of certain fractures (e.g., ankle) but with a higher risk of suffering other fractures (e.g., wrist) [90,91,92]. Increased bone mineral density (BMD) in obesity is related to the combination of a higher mechanical load and hormonal activity. However, there are other genetic and environmental factors that can also influence bone mass in obese patients, such as tobacco consumption, eating habits, and physical activity [26]. Although in older populations, it seems that obesity protects bone, it is not entirely clear whether excessive fat accumulation has beneficial or detrimental effects on bone health [83]. In fact, some evidence points out that obesity may be harmful to bone [26] due to a combination of factors, including an increase in bone marrow adipogenesis at the expense of osteoblastogenesis and adipokine (leptin and adiponectin) dysregulation [93]. In addition, increased body fat has been associated with the secretion of proinflammatory cytokines (TNF-α, IL-6, C-reactive protein) that may affect bone remodelling and could be detrimental for bone health [27,93,94,95]. Data from experimental animal models also demonstrate negative effects of high-fat diets on bone metabolism [27]. Mice fed with a high-fat diet had decreased trabecular bone volume and cortical bone growth [96]. High-fat diet also reduces BMD and promotes infiltration of adipocytes in the bone marrow [97]. In addition, cholesterol and its metabolites, which are usually elevated after high fat intake, are known to have effects on bone homeostasis through regulating differentiation and activation of osteoblasts and osteoclasts [98]. Thus, hypercholesterolemia may reduce bone mineral content (BMC) and trabecular structural parameters [99,100]. A recent study conducted in male Wistar rats fed with high-carbohydrate, high-fat diet to induce metabolic syndrome showed a deterioration in trabecular bone structure evidenced by a significant decrease in osteoblast and osteoid surfaces, osteoid volume, and significantly increased eroded surface. However, no changes in whole body BMC and BMD were found [101]. Other studies have reported both an increase [102] and a reduction [103] in BMC and BMD in rats fed with diets that induce metabolic syndrome. It has been proposed that differences in dietary fatty acids might reflect different effects on bone tissue [101]. Epidemiological studies in humans show inconclusive results on the relationship between metabolic syndrome and bone health status [104]. 

**Figure 4:**
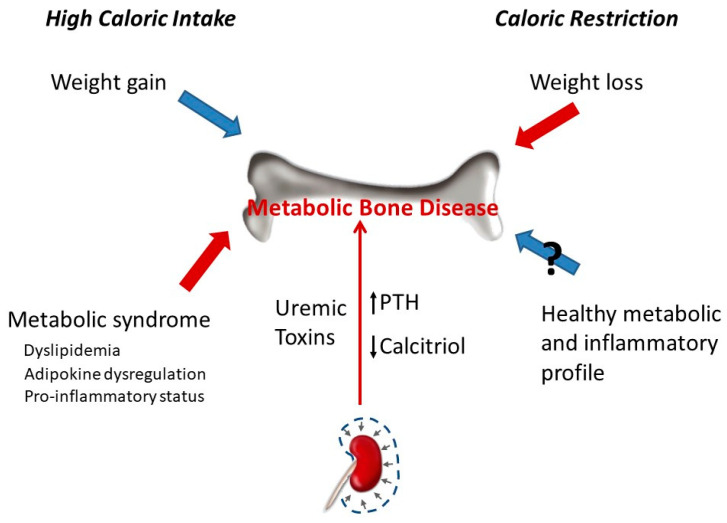
Chronic kidney disease leads to metabolic bone disease due to direct action of uremic toxins on bone and to associated changes in mineral metabolism (e.g., decreased calcitriol, increased parathyroid hormone (PTH)). The effect of calorie intake on bone can be differentiated in two types of mechanisms: mechanical stress associated with body weight and metabolic/inflammatory changes. The gain in body weight secondary to high calorie intake promotes bone anabolism due to increased mechanical load, and the opposite happens when body weight is lost after calorie restriction. However, the metabolic and inflammatory changes associated with high calorie intake are deleterious for bone, while the healthier metabolic and inflammatory profile observed after calorie restriction may (?) help to preserve bone mass.

Weight loss induced by CR is associated with lower BMD [105,106] but does not seem to result in decreased bone quality assessed by trabecular bone microarchitecture and the surface-to-curve-ratio [107]. In fact, it has been suggested that the reduction in bone mass after CR may not be detrimental because the reduced bone mass is commensurate to reduced body weight [108]. Weight loss in obese and overweight patients was associated with a significant decrease in volumetric BMD, cortical thickness, and estimated strength [109]. An observational study involving 1723 older men reported that weight loss was associated with lower peripheral bone strength and total BMD [110]. Perhaps, the key to this controversy is whether weight loss is achieved only through CR or is also accompanied by physical exercise. A recent study has demonstrated that, while CR was detrimental to bone structure, when CR was combined with exercise it was effective in decreasing adiposity and mitigating bone structural deterioration associated with weight reduction in obese rats [111]. On the other hand, CR has been shown to have important effects on inflammation, insulin resistance, and cardiovascular disease, all known risk factors for osteoporosis and fractures [112,113,114,115] (Figure 4).

Most studies on the influence of the caloric content of the diet on bone have been carried out in individuals with normal renal function. Thus, there is a need for more data on the effects of CR on the development and progression of CKD-MBD and its relationship with bone status. As previously mentioned, decreased BMD with increased osteoporosis and fracture risk may be complications of CR. In addition, in CKD patients, there is also an increased risk for osteoporosis and fracture risk associated with MBD. Bearing in mind that bone pathophysiology in CKD patients is quite different from healthy individuals, until specific studies are conducted to clarify the impact of CR on bone health in the scenario of CKD, CR should be considered with caution in CKD patients [8]. Moreover, when planning CR in a CKD patient, mostly in the earlier CKD stages, it would be necessary to assess bone status and to follow up the progression of bone parameters (e.g., BMD) in order to adjust caloric intake based on their evolution

In conclusion, scientific evidence about the effect of caloric intake on bone in the context of CKD-MBD is insufficient to draw definitive conclusions. However, from the available data, it would seem reasonable to be careful with energy restriction, especially in advanced stages of the disease, because it could have a negative impact on the skeleton.

## 5. Caloric Intake and VC

VC refers to mineral deposition in the vascular system, in the form of Ca-P complexes [116]. Although VC is part of the normal aging process, some diseases such as diabetes, hypertension, and CKD can accelerate this process [116,117,118]. Vascular and other soft-tissue calcifications are a major problem in end-stage CKD. In CKD patients, medial artery calcification is the most prevalent form of VC and is associated with increased stiffness of the artery wall [119]. VC is one of the major contributors to cardiovascular mortality in these patients [116,118]. 

Different studies suggest that VC, as well as bone remodelling, is regulated by inductive and inhibitory processes. During pathological conditions, the balance between pro-calcification and anti-calcification mediators in the arteries is upset and leads to ectopic mineralization [109]. Hyperphosphatemia, due to the inability to eliminate P in uremic patients, promotes VC through several mechanisms, including increased serum CaxP product, and phenotypic transdifferentiation of vascular smooth muscle cells to osteochondrogenic cells [119].

**Figure 5:**
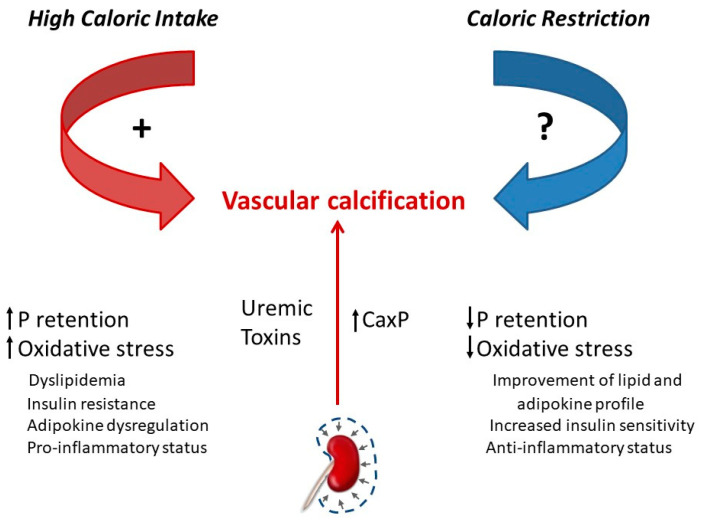
Chronic kidney disease leads to uremic vascular calcification due to increased CaxP product and to a variety of alterations related to uraemia. High caloric intake has been shown to potentiate (+) uremic vascular calcification by several mechanisms including P retention and increased oxidative stress. Although calorie restriction results in metabolic changes opposite to those elicited by high caloric intake, its influence on calcification is not clear (?) because experimental studies have failed to demonstrate a protective effect of calorie restriction on vascular calcification.

Obesity and the caloric content of the diet influence uremic VC [78,120]. In experimental models of uraemia, obese Zucker rats have been shown to develop more severe VC than lean Zucker rats. Although the mechanisms are likely to be multifactorial, oxidative stress seems to play a major role in the enhancement of VC by obesity [78]. Feeding high-calorie diets has also been shown to promote VC in non-obese uremic rats both by influencing P retention, secondary to renal α-klotho and FGF23 dysregulation, and by promoting chronic inflammation and oxidative stress [31,121] (Figure 5).

CR has been reported to improve cardiovascular health and retard vascular aging. CR attenuates the production of reactive oxygen species (ROS) and age-related increases in oxidative stress [117]. CR in young rats also prevented age-related pro-inflammatory changes in the arterial wall and preserved a more youthful aortic wall phenotype [122]. However, a recent study has failed to demonstrate a beneficial effect of CR on uremic VC. Although CR resulted in metabolic changes that could theoretically be useful to protect against VC (decrease in serum concentrations of P, glucose, lipids, leptin, etc.), it did not prevent or ameliorate VC in uremic rats [11]. Interestingly, in this study, CR resulted in increased mortality in uremic rats and an excellent direct correlation was found between body weight and survival. In these experiments, the rats subjected to CR were not obese and their reduction in body weight may have made them more vulnerable to CKD. Obesity is a risk factor to develop de novo CKD but improves survival in patients with advanced CKD, in which mortality is inversely proportional to body weight and body mass index. This is known as the obesity paradox [123]. Moreover, weight loss is associated with a poor outcome in CKD patients, so the decrease in energy stores could explain the higher mortality found in this experiment [11,19].

In summary, although high caloric intake and obesity have been shown to promote VC in experimental models, the role of CR on the induction of VC in animals is not clear, and therefore, current knowledge does not support CR as a strategy to prevent VC. Nonetheless, CR may be useful in many ways to the CKD patient, and the clinical decision about implementing CR should take into consideration the multiple facets of CR, not only related to mineral metabolism but also to progression of renal disease and mortality. The available data suggest that, in earlier stages of CKD, CR and low body weight could be beneficial, while in more advanced stages, the reduction in body weight that follows CR could increase mortality.

The interaction between energy metabolism and mineral metabolism is an exciting new field, and gaining knowledge in this area may provide a better understanding of CKD-MBD as well as opening new ways of treatment. However, the information currently available is really sparse and has been obtained mostly in animal studies; thus, more data need to be collected in humans. Some areas for future research in this field would include clarifying the relationship between energy intake and mineral metabolism at the different stages of CKD, determining the turning point at which the benefits of CR disappear and are counterbalanced by the problems associated to weight loss, examining the practicality of CR at different stages of CKD, and determining the optimal balance between caloric intake/body weight and bone health in the CKD-MBD patient.

## 6. Conclusions

Caloric intake has a significant impact on mineral metabolism and adjusting energy intake may help in the management of CKD-MBD. High caloric intake promotes P retention, while low caloric intake tends to decrease P balance. Moreover, current knowledge indicates that even for a fixed P intake, CR would help to decrease P load. Reduction in caloric intake may also aid in the control of FGF23 and calcitriol concentrations in CKD-MBD patients: high caloric intake increases FGF23 and decreases calcitriol, while the opposite changes are observed with low caloric intake. Scientific evidence about the effect of caloric intake on bone in the context of CKD-MBD is insufficient to draw definitive conclusions. However, from the available data, it would seem reasonable to be careful with CR because it could have a negative impact on the skeleton. Finally, while energy-rich diets have been shown to promote VC, there is no scientific proof demonstrating a beneficial effect of CR on soft-tissue mineralization. In conclusion, (a) when planning caloric intake for a renal patient, it is important to take into consideration the associated changes in mineral metabolism, (b) reducing calorie intake could be an additional strategy for controlling mineral disorders in CKD patients, especially in the earlier stages when weight loss/malnutrition are not yet a problem.
